# Unlocking the Cervix: Biological Mechanisms and Research Gaps in Preterm Birth

**DOI:** 10.7759/cureus.72835

**Published:** 2024-11-01

**Authors:** Joana Félix, Carla Bartosch, Alexandra Matias

**Affiliations:** 1 Obstetrics and Gynaecology, Hospital Pero Hispano, Matosinhos, PRT; 2 Obstetrics and Gynaecology, Faculdade de Medicina da Universidade do Porto, Porto, PRT; 3 Pathology, Instituto Português de Oncologia Francisco Gentil Porto, Porto, PRT; 4 Obstetrics and Gynaecology, Hospital São João, Porto, PRT

**Keywords:** cervical extracellular matrix, inflammatory response, labour genetic predisposition, molecular mechanism of cervical remodelling, preterm labour

## Abstract

Preterm birth (PTB), defined by the WHO as delivery before 37 completed weeks of gestation, remains a significant global health challenge and the leading cause of neonatal mortality. Despite extensive efforts to prevent PTB, rates have remained stable, largely due to an incomplete understanding of its underlying pathophysiology. While research has traditionally focused on the myometrium and foetal membranes, the cervix's critical role in maintaining pregnancy and initiating labour is increasingly recognized but still often underexplored. This review aims to consolidate current knowledge on cervical function and remodelling during pregnancy and labour while identifying key research gaps and potential intervention strategies. A comprehensive literature review spanning 50 years was conducted using PubMed, identifying 114 studies that met the inclusion criteria. These studies examined cervical function during labour, spontaneous PTB, and the molecular mechanisms involved in cervical remodelling (CR). The review highlights significant molecular differences in cervical remodelling between term and preterm births. Critical processes such as extracellular matrix (ECM) remodelling, immune cell activity, and genetic predispositions vary depending on the specific triggers and gestational age at the onset of labour. Inflammatory responses are especially heightened in preterm births, particularly those driven by infections. However, much of the current data are derived from animal models, which may not accurately represent human physiology. In conclusion, a deeper understanding of the complex molecular mechanisms driving cervical remodelling is crucial to addressing PTB. Future research should prioritize human tissue studies to bridge the gap between animal models and human physiology. Developing targeted interventions that address cervical insufficiency and improve pregnancy outcomes is essential for reducing PTB rates.

## Introduction and background

Background

Preterm birth (PTB) is defined by the World Health Organization (WHO) as birth before 37 completed weeks of gestation or fewer than 259 days from the first day of a woman’s last menstrual period [1]. The global prevalence of PTB is estimated at around 9.9%, with significant regional variations and a higher burden in low- and middle-income countries [2-4].

PTB is a major concern due to its severe impact on neonatal mortality and long-term health complications, resulting in approximately 3.1 million neonatal deaths annually. It is associated with acute conditions such as respiratory distress syndrome and necrotizing enterocolitis, as well as chronic disorders, including cardiovascular and neuropsychiatric issues [5-8]. Beyond immediate neonatal care, PTB imposes substantial economic and healthcare costs and perpetuates a generational cycle of preterm births [6,9,10].

While global health initiatives, including the Millennium Development Goals, have aimed to reduce preterm birth rates, progress has been limited [11,12]. PTB is a multifactorial syndrome with many underlying causes, such as infections, maternal health conditions, and genetic factors, which makes it difficult to address with a single approach. Additionally, the aetiology of PTB is still not fully understood, which hinders the development of effective screening methods and therapeutic interventions. Ongoing research is crucial to better understand the diverse mechanisms behind PTB and to develop targeted, successful strategies.

Addressing PTB is challenging due to its multifaceted causes [13]. Research from the 1990s identified premature cervical remodelling (CR) as a key factor in PTB risk [14]. Romero et al. highlighted cervical ripening as a hallmark of "premature parturition syndrome," emphasizing CR as a primary molecular event in PTB [15]. This underscores the cervix’s crucial role in PTB development and suggests that targeting CR could be a promising approach for screening and intervention strategies. This review aims to summarize current knowledge of cervical function in both normal and premature labour and its role in triggering PTB, while identifying critical gaps for future research.

## Review

Methods

Our literature review examined clinical studies, systematic reviews, and literature reviews on the role of the cervix in labour, particularly premature labour. We searched the PubMed database for full-text studies published in English or Portuguese over the past 50 years, using the search terms: "role of cervix in spontaneous preterm birth," "cervix in labour," "cervical biopsies in labour," and "cervical biopsies in preterm labour."

The inclusion criteria encompassed studies investigating the function of the cervix during labour, research addressing the role of the cervix in spontaneous preterm birth, animal studies exploring cervical roles in preterm birth, and studies utilizing cervical biopsies to examine molecular and cellular processes during cervical remodelling in labour. In contrast, we excluded articles focused on multiple pregnancies, research on induced preterm birth, studies investigating preterm birth with known causes (e.g., infection, hydramnios, foetal malformation), and biopsy studies primarily aimed at detecting cervical lesions.

**Figure 1:**
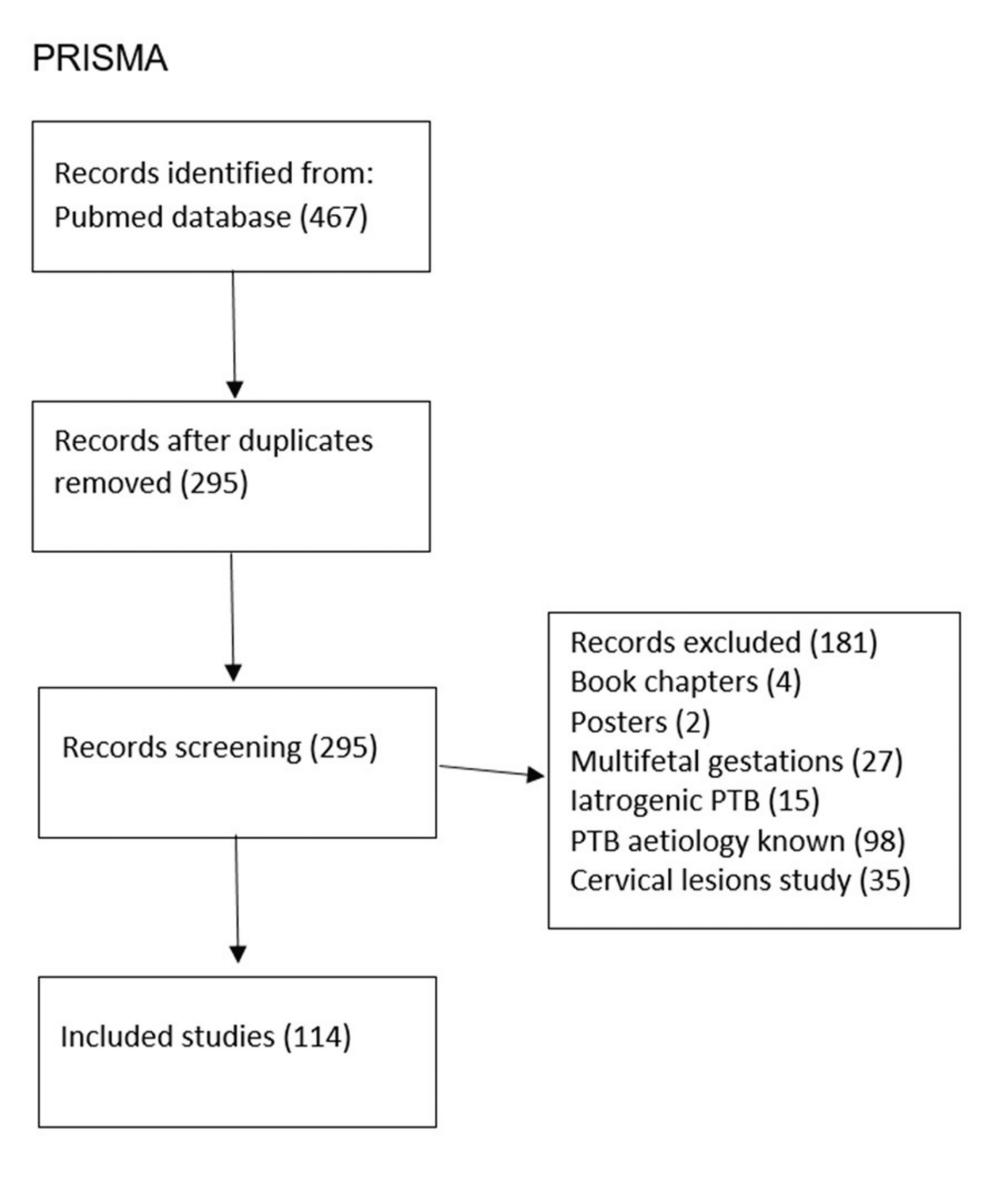
Study flowchart (PRISMA)

Initially, 467 articles met the inclusion criteria and underwent abstract review. After applying the exclusion criteria, the final number of articles included in the review was 114 (Figure 1).

Cervical function, structure, and remodelling during pregnancy

The cervix acts as a vital mechanical barrier throughout pregnancy, remaining closed until term and undergoing substantial remodelling for successful delivery. Understanding CR is crucial, as deviations from normal patterns increase the risk of PTB. Despite extensive research, knowledge of cervical tissue mechanics remains limited due to the challenges of studying cervix samples during pregnancy, a period often considered a "protected state." Advances in animal studies, imaging technologies, and molecular biology have offered valuable insights into these processes and their implications for pregnancy outcomes.

The cervix is composed of multiple tissue layers, each with a specific function. Its epithelial outer surface, facing the vagina, is lined with stratified squamous epithelium, which protects it from physical trauma and infections. The inner epithelial portion, the endocervix, is lined with columnar epithelial cells that secrete mucus, which plays a key role in fertility by controlling the passage of sperm [16,17]. Beneath these epithelial layers lies the stroma, a structure made up of dense extracellular matrix (ECM) and various cell types. The ECM, consisting of fibrillar collagens, elastin fibres, proteoglycans, glycosaminoglycan’s (GAGs), and matricellular proteins, is essential for the cervix's mechanical properties and structural integrity [18-20]. Cells such as fibroblasts, smooth muscle cells, immune cells, glandular cells, and vascular cells each play specialized roles in matrix production, tone regulation, and immune response [16,21]. Additionally, the cervix is highly vascularized and innervated, ensuring adequate blood flow and sensory function. Notably, unlike the rest of the uterus, the cervix exhibits increased innervation throughout pregnancy [22]. 

Traditionally, the cervix has been viewed as a homogeneous structure, with the ECM comprising 85-90% of its content and cells contributing only 10-15% [20,23,24]. However, recent research has revealed a more intricate and heterogeneous architecture, with regional variations in collagen dynamics and cellular composition [23-25]. Optical coherence tomography has highlighted the presence of circumferential collagen fibres in the middle stroma, which are crucial for maintaining cervical integrity and resisting dilation [22]. Additionally, differences in collagen cross-linking and smooth muscle distribution, especially at the internal os, suggest specialized functions and higher contractility in these regions [25-29]. This evidence underscores the need to recognize location-specific differences in cervical architecture and cell distribution, as these significantly influence the cervix’s role during pregnancy and labour [20].

During pregnancy, the cervix serves a dual function: it acts as a formidable barrier against microbial invasion and mechanical stress from the expanding uterus while also undergoing a controlled softening and dilation process to facilitate childbirth. This delicate balance is essential for ensuring a healthy pregnancy and safe delivery [17,29-31]. Cervical remodelling during pregnancy occurs in four overlapping phases: softening, ripening, dilation, and postpartum repair. Softening increases the cervix’s pliability, followed by ripening, which enhances its compliance in preparation for dilation. After childbirth, the cervix undergoes postpartum repair to restore its function for future pregnancies [22,31-33]. Effective cervical remodelling is vital for delivery, as uterine contractions alone cannot facilitate labour if the cervix remains rigid [17,23,26,33-37].

Cervical softening is the initial phase of this transformation, marked by changes in tissue properties, such as reduced stiffness and increased compliance, while maintaining tissue integrity [18,25]. Early signs of softening, driven by cellular proliferation and matrix remodelling involving fibroblasts, myofibroblasts, and epithelial cells, can appear as early as one month after conception [17,18,27,32]. Recent research has explored several biomarkers and imaging methods for detecting early cervical changes linked to preterm birth risk. Biomarkers like foetal fibronectin (fFN), IL-6, and matrix metalloproteinases (MMPs) show promise as early indicators due to their association with cervical remodelling and inflammation [19,38,39].

For imaging, transvaginal ultrasound remains the primary non-invasive tool for assessing cervical length (CL), a key predictor of preterm birth. Also, elastography has emerged as a promising technique for assessing cervical stiffness, detecting early softening before significant shortening occurs [40,41].

At this stage, architectural changes in the ECM are characterized by increased collagen turnover and reduced cross-linking. As pregnancy progresses and matures, cross-linked collagen is gradually replaced by less cross-linked forms, facilitating cervical softening while maintaining structural integrity [20,25-27]. During this process, collagen type I fibres evolve from long, thin structures to thicker, curved forms, though the overall content of collagen type I remains stable [20,21,25-27,29,30]. Additionally, cervical epithelial cells play a critical role in providing immunoprotection and maintaining the barrier to safeguard the stroma during ECM remodelling [21].

Following softening, cervical ripening is critical for preparing the cervix for dilation. This phase involves structural changes such as an elevated Bishop Score and a reduced ability of the cervix to maintain closure as a load-bearing organ [18,42-44]. ECM changes during ripening include altered collagen cross-linking, increased fibre diameter, and shifting from straight to wavy fibres [20,21]. GAGs, particularly hyaluronic acid (HA), play critical roles in collagen packing and tissue hydration, with HA levels rising due to oestrogen-driven upregulation of hyaluronan synthase (Has) 2 [17,21,32,42,45,46]. This increase in HA solubilizes collagen, triggering an inflammatory response that is crucial for ripening. Elevated proteoglycans also regulate ECM organization, influencing cellular adhesion and proliferation [17,47].

In the 1980s, Mont Liggins proposed that immune cell infiltration during cervical ripening leads to ECM degradation and remodelling. However, this idea has since been challenged, as it was based on studies using postpartum biopsy samples, which more likely reflect tissue repair mechanisms after childbirth rather than the pre-labour ripening process [21,29,42,48,49]. Recent research suggests that inflammatory cells and matrix metalloproteinases (MMPs) primarily aid in active dilation and postpartum repair rather than initiating ripening [17,21,29,42,47,48,50-53]. Current evidence indicates that cervical ripening is mainly driven by collagen reorganization through increased GAG synthesis rather than an inflammatory response [17].

Epithelial cells also contribute to ripening by providing barrier protection and facilitating enzymatic activity [54,55]. This process involves controlled adjustments in barrier properties, hydration levels, and intercellular communication [56-58]. Reversible epithelial-mesenchymal transition (EMT) and its counterpart, mesenchymal-to-epithelial transition (MET), regulate cellular remodelling in both the amnion and cervix, driven by steroid hormones during gestation [21,31]. Research suggests that oestrogen promotes EMT, cellular migration, and cervical inflammation, while progesterone supports tissue repair and counteracts infection or inflammation in the cervix [59-64].

The final stage, dilation, is often difficult to distinguish from ripening, as the two phases overlap [17,18,21,32,42]. At term, increased macrophage and neutrophil densities and higher levels of proinflammatory cytokines are associated with cervical dilation [16,17,29,32,65]. Structural changes such as collagen degradation and altered cell density further characterize this phase [29].

Premature cervical remodelling as a contributing factor to preterm birth

Recent research by Romero et al. has highlighted cervical ripening as a critical component of what is termed “premature parturition syndrome.” They noted that PTB frequently involves early changes in gestational tissues occurring weeks before labour onset. Specifically, signs of cervical ripening-such as cervical shortening and internal os patency-can be detected through vaginal examinations and ultrasound imaging weeks before a spontaneous preterm labour [17,33,34,37,66-76]. Understanding early cervical changes provides a crucial opportunity for preventing preterm birth by enabling the identification of high-risk patients through biomarkers or advanced imaging techniques. Early detection allows clinicians to intervene before the cervix reaches a critical stage of ripening, offering the chance for timely interventions, such as progesterone or cervical cerclage. This knowledge also highlights a sequence of physiological events that could be targeted for screening and intervention, potentially improving pregnancy outcomes [15,33].

Initially described as "precocious cervical ripening" by Papiernik et al. in 1986, the phenomenon has evolved into the concept now recognized as "premature cervical remodeling." This term encompasses a range of processes, including softening, shortening, and dilation of the cervix [34,77]. Research demonstrates an inverse relationship between CL and gestational age at birth, which challenges the traditional view of cervical incompetence (CI) as a binary condition [34,37,67-72,78-80]. Recognizing and monitoring these early cervical changes can provide a crucial window for screening and identifying women at increased risk of preterm labour. However, to devise effective interventions, further exploration into the underlying pathophysiology of premature cervical remodelling and the implications of a shortened cervix is essential [15,81].

A multitude of factors have been associated with a short cervix, including the cessation of progesterone action, congenital cervical hypoplasia, history of previous cervical surgeries, and infections. Despite these associations, the precise mechanisms contributing to cervical "failure" and the resulting development of a sonographically short cervix remain poorly understood [80,82-86]. Key challenges in elucidating cervical “failure” include the lack of consistent definitions, the scarcity of robust human data, and methodological discrepancies in research. These discrepancies may arise from variations in biopsy locations, timing, and the presence of inadequate control groups [22,87]. This review aims to address these critical issues by synthesizing and examining evidence from both animal models and human studies. Through this approach, we hope to clarify the known mechanisms underlying premature cervical remodelling while identifying gaps in knowledge. This will enable us to design studies that enhance our understanding and explore novel screening methods and treatment strategies that could be implemented to improve outcomes for at-risk populations.

Molecular mechanisms linked to premature cervical remodelling: insights from animal studies

Much of our understanding of labour, both physiological and premature, has been derived from animal models due to the challenges in obtaining human cervical samples during pregnancy. Research utilizing these models has provided valuable insights into the mechanisms of labour, bridging theoretical knowledge with practical applications in human obstetrics. However, while animal models offer valuable insights into the biological mechanisms underlying cervical changes, their findings may not fully translate to human physiology. For example, in mice, labour is linked to a decline in progesterone levels, a phenomenon that is not observed in humans. It is essential to recognize the significant differences in reproductive anatomy, hormonal regulation, and gestational timelines. Therefore, caution is warranted when extrapolating findings from animal models to human contexts. A thorough understanding of both the similarities and differences between species is crucial before drawing direct correlations (Table 1) [66,88-93].

**Table 1:** Advantages and limitations of animal models in cervical remodelling and preterm birth research

Animal model	Advantages	Limitations
Rodents	*Cost-effective*, can study large sample sizes, *short gestation period*, with rapid data collection and genetic manipulation tools available.	Significant anatomical and hormonal differences from humans
Sheep	Similar cervical structure and reproductive physiology to human, larger size facilitates surgical interventions and tissue collection.	*Higher cost*, *complex management*, and fewer tools for genetic modifications.
Non-human Primates	Closest to human reproductive biology	High cost of maintenance and ethical considerations; fewer animals available for studies.
Guinea pigs	Similar cervical changes to humans	Longer gestation periods than rodents; different reproductive physiology
Rabbits	Accessible and cost-effective	Differences in cervical structure and remodelling

ECM changes of the cervix can manifest through various processes, depending on the triggers that lead to ripening. For instance, RU486 (mifepristone) models show accelerated cervical ripening, with pronounced matrix disorganization and partially activating pro-inflammatory and repair processes akin to postpartum changes through mechanisms distinct from term ripening [21,94,95]. In contrast, lipopolysaccharide (LPS) treatment induces cervical changes and labour within hours of exposure with architectural alterations of the collagen matrix that resemble those seen in term ripening [21,95]. Additionally, the expression of matrix synthases differs under these conditions, with HAs two genes increasing near term, while Has1 expression rises in preterm scenarios [17,32,95].

Prostaglandin expression plays a critical role in cervical remodelling, as they reduce cervical resistance by reorganizing collagen fibrils [96]. In models of premature parturition, the expression of prostaglandin-cyclooxygenase-endoperoxide synthase (Ptgs) is upregulated, with Ptgs1 levels heightened in RU486-induced ripening and Ptgs2 activated in LPS models [95]. Non-steroidal anti-inflammatory drugs, which inhibit prostaglandin production, have been shown to effectively reduce both term and preterm labour in animal studies. Research indicates that cyclooxygenase (COX) 1 activity is essential for timely term labour in mice, whereas COX 2 expression is induced during inflammation-mediated preterm labour [97,98].

Cellular responses vary significantly depending on the trigger that induces labour. In the context of term cervical ripening, neutrophil depletion does not affect the timing or success of parturition, and general inflammatory responses typically begin only after labour has commenced [29,50]. In a transgenic mouse model, cervical ripening did not occur despite the infiltration of neutrophils and macrophages, suggesting that neutrophil activation is not essential for term cervical ripening and that merely having inflammatory cells may not be sufficient to initiate this process [99]. In contrast, RU486-induced premature cervical ripening is associated with a marked increase in tissue monocytes and eosinophils compared to term conditions, while LPS-induced ripening is characterized by elevated neutrophil levels without corresponding changes in monocyte or eosinophil counts [95]. The precise role of eosinophils remains unclear; however, in rodents, an increase in eotaxin-1 transcripts is followed by a rise in eosinophils on the day of birth, indicating that eosinophils may facilitate the differentiation of monocytes into alternatively activated macrophages during postpartum repair [21,42].

Cytokine expression also varies between term and preterm births. In terms of labour, cytokines tend to increase postpartum rather than during labour itself [58]. Conversely, in preterm models, there is a significant rise in the gene expression of neutrophil chemoattractant Cxcl2 and other pro-inflammatory cytokines, with LPS-induced inflammation showing a greater increase than that induced by RU486 [95,100].

Complement activation also seems to play a significant role in labour. Two mouse models of PTB have highlighted the importance of complement activation in CR and the onset of PTB. Findings from these models demonstrated increased deposition of cervical complement component (C) 3 and elevated levels of C5a in PTB cases compared to gestational age-matched controls. Furthermore, C5a receptor (C5aR)-deficient mice failed to exhibit cervical remodelling or experience PTB in response to treatments with LPS or mifepristone. This strongly suggests that C5aR is essential for the cervical remodelling process that precedes preterm birth [44,101].

Hormonal influence plays a pivotal role in the processes associated with labour and cervical remodelling. In rodent models, the onset of labour is characterized by a significant decline in maternal progesterone levels, indicating its crucial role in parturition [102-104]. Conversely, multiple animals and in vitro studies have underscored the potential of oestrogen in facilitating cervical ripening, labour, and delivery [105,106]. Increased expression levels of oestrogen receptor α in the cervix have been associated with spontaneous preterm labour and delivery, highlighting oestrogen influence in this context [105-109].

Epithelial cell lesions and disruptions in barrier protection are also associated with premature cervical remodelling. A study utilizing a mouse model demonstrated that the combination of vaginal bacteria and mild cervical injury from a spermicide, which causes cellular damage, led to higher rates of preterm birth compared to exposure to bacteria alone. In contrast, the administration of vaginal progesterone modified the junctional proteins of the cervical epithelium and significantly reduced LPS-induced preterm birth [110-112]. This highlights the critical role of the cervical epithelium in maintaining barrier function against infections and underscores its importance in preventing preterm birth.

Neuromodulation and sensory nerve activity significantly influence reproductive tissue dynamics and cervical remodelling. When stimulated by microorganisms or toxins, sensory nerves in rats release neurotransmitters, initiating local inflammatory responses known as neurogenic inflammation [113,114]. Moreover, oestrogen can stimulate the production of neuropeptides from cervical sensory neurones, which are increased in the cervix during pregnancy, highlighting the intricate interaction between the endocrine and nervous systems [115].

Mechanical forces have recently emerged as a crucial factor in the intricate process of cervical remodelling. Research highlights a strong correlation between mechanical stimuli and biochemical signals, which can lead to significant changes in cellular behaviour and the extracellular matrix. Utilizing proteome-wide technology, studies have investigated the mechano-related signalling molecules present in the mouse cervix, providing valuable insights into how physical forces influence cervical modifications during pregnancy. These investigations have identified an increase in various cytoskeletal components and signalling molecules that correlate with the escalating gravitational forces exerted by the growing foetus on the cervix. This finding underscores the notion that foetal size and weight are not merely passive characteristics; rather, they may play a pivotal role in cervical remodelling through mechano-transduction processes [31].

Molecular mechanism linked to premature cervical remodelling: what we know?

The process of labour is inherently complex and multifactorial, involving a myriad of physiological and biochemical interactions. Given this complexity, it is not surprising that multiple molecular processes may lead to premature CR and subsequently contribute to the risk of PTB. Understanding the molecular mechanisms behind premature CR is crucial for addressing this critical risk factor. Disruptions at both cellular and molecular levels can compromise cervical integrity and trigger preterm labour, influenced by factors such as cellular dysfunction, inflammation, environmental influences, and genetic predispositions.

Cervical epithelial dysfunction compromises the essential barrier that prevents microorganisms from ascending from the vaginal canal to the uterine cavity. Maintaining this barrier is vital during CR, as any compromise can significantly elevate the risk of PTB. Various factors, including cervical surgeries, infections, inflammation, genetic predispositions, and chemical exposure, disrupt this protective barrier, ultimately leading to PTB [16,66,81,110,111].

Reversible EMT, along with its counterpart, MET, orchestrates cellular remodelling in both the amnion and cervix, contributing to the dynamic changes observed during gestation [62,116]. This cyclic remodelling process is intricately regulated by steroid hormones: oestrogen promotes EMT and cervical inflammation, while progesterone supports tissue repair and mitigates adverse effects [59-62]. Disruption of this cycle, triggered by infections or other stressors, can lead to a non-reversible state of EMT, resulting in chronic inflammation and degradation of the cervical matrix. This, in turn, compromises cervical integrity and accelerates premature remodelling [16,29,31,45,47,62-64,116]. These findings underscore the importance of EMT and epithelial functions in maintaining barrier properties and immune surveillance [42,81,116].

Inflammation at the maternal-foetal interface is a critical and common factor associated with PTB. Prolonged cervical inflammation can lead to significant tissue damage, resulting in cervical insufficiency and shortening, which frequently culminates in PTB [110,117-119]. Women diagnosed with cervical insufficiency often exhibit elevated levels of interleukin (IL)-6 in the amniotic fluid, suggesting a strong inflammatory component in these cases [120]. Inflammation can be triggered by both infectious and non-infectious stimuli. Approximately 40% of PTBs are linked to intrauterine infections, which typically originate from ascending bacteria; however, this review does not explore the specifics of these infections in detail [91,110,111,121]. On the other hand, non-infectious stimuli, such as toxic exposure, can also induce a sterile inflammatory response that leads to cervical extracellular matrix remodelling [110,117,122]. Inflammatory processes triggered by infections or non-infectious factors play a crucial role in prematurely remodelling cervical tissues.

Numerous endogenous and exogenous factors contribute to the complex phenomenon of premature CR, yet our understanding of these processes remains incomplete. One significant factor is oxidative stress, which can arise from various sources, including environmental pollutants such as cigarette smoke. A delicate balance between reactive oxygen species (ROS) and antioxidants is essential for maintaining tissue integrity and ensuring optimal maternal-foetal health. When this balance is disrupted, it can lead to premature cellular senescence, chronic inflammation, and an elevated risk of PTB. The specific role of oxidative stress in conditions such as cervical incompetence and short cervix remains somewhat inconsistent, warranting further investigation [117,122]. Cervical trauma and anatomical malformations have also been implicated in the risk of preterm birth [85,123-126]. Endocrine factors, particularly the balance between progesterone and oestrogen, also affect the cervix function [21,31,62]. Furthermore, mechanical factors, including foetal weight and the presence of mechano-sensitive molecules, contribute to the processes of premature cervical remodelling [31]. Understanding how these diverse factors interact and the respective pathways leading to premature cervical remodelling is crucial for developing effective prevention strategies against preterm birth.

Genetic predisposition is another critical element in cervical remodelling. While cervical trauma and inflammation contribute to CI, genetic predispositions explain why only some women with these exposures develop CI and subsequent PTB [123]. Specific polymorphisms in genes involved in connective tissue metabolism and inflammatory responses, such as collagen 1A1, transforming growth factor beta, and the interleukin-10 gene, have been associated with an increased risk of PTB due to their role in disrupting connective tissue regulation [21,78,123,127]. Furthermore, genetic anomalies that affect collagen and elastin synthesis, as observed in conditions like Ehlers-Danlos syndrome and Marfan syndrome, further elevate susceptibility to CI [21,128].

Biomarkers reflect physiological changes and offer valuable insights into the pathways leading to preterm birth [19]. Extensive research has been conducted on various biological fluids to identify effective indicators. Extracellular matrix degradation-related biomarkers, such as fFN, have shown particular promise. The presence of fFN in cervicovaginal fluid after the 24th week of pregnancy indicates matrix disruption and an increased risk of spontaneous preterm birth (sPTB). In clinical practice, fFN is recognized for its high negative predictive value (NPV), making it a valuable tool in predicting preterm labour [129,130]. Inflammatory markers also play a significant role in cervical changes and can serve as potential screening tools for PTB. For instance, IL-6 is associated with cervical remodelling, while other markers like IL-8 and C-reactive protein (CRP) have demonstrated promise in predicting PTB [19,131,132]. Despite the various biomarkers studied, their relevance to early cervical changes necessitates further investigation.

The interplay between endogenous and exogenous factors such as infections, chemical exposures, mechanical damage, oxidative stress, and endocrine disruptions intersects genetic predispositions to influence premature cervical remodeling. This multifactorial nature of PTB emphasizes that CR involves various pathways influenced by different triggers. Notably, the molecular mechanisms governing CR vary significantly between preterm and term births, as well as among different causes of PTB (Table 2). Understanding both normal and abnormal CR processes is crucial for identifying individuals at risk and developing targeted interventions to mitigate preterm birth linked to cervical insufficiency.

**Table 2:** Molecular mechanisms linked to premature cervical remodelling. CR: cervical remodelling; EMT: epithelial-mesenchymal transition; MMP: matrix metalloproteinases; GAG: glycosaminoglycan.

Molecular mechanism	Impact on premature cervical remodelling
Cervical epithelial dysfunction	Compromises the cervix’s barrier function, increasing susceptibility to infections and inflammation.
Inflammation	Involves immune response activation and cytokine release. Chronic inflammation accelerates CR, causing cervical shortening and insufficient cervix.
Oxidative stress	The imbalance between reactive oxygen species and antioxidants contributes to premature CR by inducing cellular senescence and sterile inflammation.
Hormonal disruption	Hormonal imbalances can precipitate the disruption of normal softening and ripening processes. Oestrogen enhances EMT, cellular migration, and cervical inflammation.
Genetic predisposition	Genetic variations in genes related to connective tissue metabolism (e.g., TGF-β, IL-10) increase sensibility to environmental factors.
Matrix metalloproteinases	Altered MMP activity can lead to abnormal matrix remodelling and premature CR.
Glycosaminoglycan	Changes in GAG composition and metabolism affect matrix hydration and collagen organization and CR.
Epithelial-mesenchymal transition	Persistent EMT can disrupt cervical barrier function and stimulate inflammations, promoting early remodelling
Complement activation	Increased complement activation enhances inflammatory responses and matrix remodelling and can accelerate CR.
Prostaglandins (PGs)	Upregulation of prostaglandins can enhance cervical matrix remodelling and inflammation, leading to premature CR.
Mechanical forces	Mechanical stress can induce CR by activating mechano-sensing pathways in cervical tissues.

Clinical implications and future directions

Recent animal studies have highlighted key differences in cervical remodelling between term and preterm births. Term remodelling is primarily characterized by collagen disorganization, while preterm remodelling involves an influx of inflammatory cells, such as neutrophils and mononuclear cells. This distinction is critical, as it suggests that different molecular and cellular pathways may be at play depending on the timing and triggers of labour [29,32,95]. However, significant gaps remain in our understanding of the biological mechanisms of labour, particularly due to the scarcity of human cervical tissue evaluations in affected women, with much of the available data based on animal models [22,32]. Additionally, many studies rely on cervical biopsies obtained postpartum, focusing more on the tissue repair phase than the active remodelling phase. Furthermore, these studies often lack adequately matched control groups in terms of gestational age or use samples from non-pregnant women, limiting the applicability of their findings.

Identifying markers for premature cervical remodelling is crucial for developing effective screening methods. Future research should focus on elucidating the molecular and cellular mechanisms underlying cervical changes, including EMT, inflammation triggers beyond infection, the effects of endocrine imbalance, the influence of toxic and exogenous factors, and the role of genetic polymorphisms. Integrating clinical and molecular insights will significantly enhance our understanding of cervical remodelling, ultimately leading to more targeted and personalized interventions for women at risk of preterm birth.

Advancements in non-invasive imaging technologies and biomarker identification present promising avenues for improving screening and monitoring capabilities. However, there is a pressing need for increased research utilizing human cervical tissue to gain deeper insights into the mechanisms of cervical remodelling and develop targeted therapies. For example, molecular studies on the behaviour of inflammatory cells, such as neutrophils and mononuclear cells, in triggering labour could help clarify how inflammation leads to preterm birth and inform the development of targeted interventions. Moreover, tracking cervical changes in high-risk populations throughout pregnancy will help identify early biomarkers that can predict premature cervical remodelling and preterm birth. It is also vital to investigate the impact of environmental exposures, lifestyle factors, and maternal health on cervical remodelling.

The main methodological limitation of this study is the use of a single research database. Additionally, the reliance on animal models and the scarcity of robust human data may limit the generalizability of the findings to human populations.

Finally, integrating the fields of obstetrics, molecular biology, genetics, and bioengineering will facilitate innovative research and clinical applications, making this research fundamental for advancing our knowledge and improving outcomes for at-risk pregnancies.

## Conclusions

The cervix plays a critical role in maintaining a healthy pregnancy, undergoing intricate remodelling processes that are essential for successful delivery. Throughout pregnancy, the cervix adapts structurally and functionally, with distinct differences observed between term and preterm birth scenarios. This remodelling is influenced by a multitude of factors, including hormonal changes, mechanical stress, and cellular interactions, all of which contribute to the cervix's ability to function effectively during labour.

Despite the progress made in understanding cervical remodelling, significant gaps persist in our knowledge, particularly regarding the specific mechanisms that govern these processes and their direct implications for preterm birth. Addressing these gaps is crucial for developing targeted interventions to prevent preterm labour. By deepening our understanding of cervical dynamics, we can enhance screening and treatment options, ultimately improving outcomes for women at risk of preterm birth.

## Appendices

Search strategy

Objective

To explore cervical function in both normal and premature labour and its role in triggering PTB

Databases Searched

PubMed

Search Terms: 

The following keywords and phrases were used to conduct the literature search:

"role of cervix in spontaneous preterm birth"

"cervix in labour"

"cervical biopsies in labour"

"cervical biopsies in preterm labour"

Inclusion Criteria

  Study Types

     Peer-reviewed original research articles

     Review articles

     Clinical trials

     Meta-analyses

     Animal studies

  Population

     Pregnant women at risk of preterm birth

     Animal models relevant to cervical remodelling and preterm birth

  Language

     English language publications

  Publication Date

     Studies published in the last 50 years to ensure current relevance, with particular emphasis on recent findings.

Exclusion Criteria

Multiple pregnancies

Studies focusing on non-pregnant populations or studies primarily aimed at detecting cervical lesions.

Studies investigating preterm birth with known causes (e.g., infection, hydramnios, foetal malformation

Research lacking a focus on cervical remodelling or preterm birth mechanisms.

Data Extraction

Abstracts and full-text articles were reviewed to extract relevant data concerning the molecular mechanisms, hormonal influences, inflammatory processes, and genetic predispositions linked to premature cervical remodelling.

Key findings related to biomarkers and potential intervention strategies were also noted.
